# Non-volatile electric control of spin-orbit torques in an oxide two-dimensional electron gas

**DOI:** 10.1038/s41467-023-37866-2

**Published:** 2023-05-05

**Authors:** Cécile Grezes, Aurélie Kandazoglou, Maxen Cosset-Cheneau, Luis M. Vicente Arche, Paul Noël, Paolo Sgarro, Stephane Auffret, Kevin Garello, Manuel Bibes, Laurent Vila, Jean-Philippe Attané

**Affiliations:** 1 Université Grenoble Alpes/CEA/IRIG/SPINTEC, Grenoble, France; 2 Unité Mixte de Physique, CNRS, Thales, Université Paris-Saclay, Palaiseau, France; 3 Department of Materials, ETH Zurich, 8093 Zurich, Switzerland; 4 Institut Universitaire de France, Paris, France; 5 Present Address: Physics of Nanodevices, Zernike Institute for Advanced Materials, University of Groningen, 9747 AG Groningen, the Netherlands

**Keywords:** Magnetic devices, Information storage

## Abstract

Spin-orbit torques (SOTs) have opened a novel way to manipulate the magnetization using in-plane current, with a great potential for the development of fast and low power information technologies. It has been recently shown that two-dimensional electron gases (2DEGs) appearing at oxide interfaces provide a highly efficient spin-to-charge current interconversion. The ability to manipulate 2DEGs using gate voltages could offer a degree of freedom lacking in the classical ferromagnetic/spin Hall effect bilayers for spin-orbitronics, in which the sign and amplitude of SOTs at a given current are fixed by the stack structure. Here, we report the non-volatile electric-field control of SOTs in an oxide-based Rashba-Edelstein 2DEG. We demonstrate that the 2DEG is controlled using a back-gate electric-field, providing two remanent and switchable states, with a large resistance contrast of 1064%. The SOTs can then be controlled electrically in a non-volatile way, both in amplitude and in sign. This achievement in a 2DEG-CoFeB/MgO heterostructures with large perpendicular magnetization further validates the compatibility of oxide 2DEGs for magnetic tunnel junction integration, paving the way to the advent of electrically reconfigurable SOT MRAMS circuits, SOT oscillators, skyrmion and domain-wall-based devices, and magnonic circuits.

## Abstract (web summary)

A central goal of spintronics is electric control of magnetism. One particularly promising method makes use of spin-orbit torques which arise due to the combination of electric current, and the intrinsic spin-orbit effect in a material. Here, Grezes et al demonstrate non-volatile electrical control of the spin-orbit torque generated at the interface between an oxide and a metal.

## Introduction

The efficient control of the magnetization using currents is the key requirement to develop high-performance spintronics devices for information and communication technology. In the last decade, current-induced magnetization switching by spin-transfer torque has become a well-established technology, with nowadays major foundries and integrated device manufacturers commercializing Spin-Transfer Torque Magnetic Random Access Memories (STT-MRAM)^1^. Meanwhile, spin–orbit coupling emerged as an alternative method to generate spin currents and to manipulate magnetization efficiently. SOTs rely on the injection of an in-plane current in a non-magnetic material adjacent to a ferromagnet. The spin–orbit coupling creates a spin accumulation at the interface that induces a torque on the ferromagnet magnetization. In heavy metal (HM)/ferromagnetic (FM) heterostructures^2,3^ SOTs arise from the bulk spin Hall effect (SHE) and/or the Edelstein effect associated with an interfacial Rashba state. These spin–orbit torques (SOTs) have attracted increasing attention, owing to their ability to induce magnetization oscillations or switching^4^, and their potential for high speed, high endurance, and low-energy switching^5^.

In recent years, oxide two-dimensional electron gases (2DEGs) have emerged as a new promising SOT system^6–8^. They display an efficient spin-charge interconversion through the direct and inverse Edelstein effects, arising from their broken inversion symmetry which induces a Rashba-type spin–orbit coupling. In previous works, we showed an enhancement of the spin-to-charge conversion efficiency by two orders of magnitude in such SrTiO_3_-based 2DEG compared to conventional HM/FM heterostructures^8^, along with a nonvolatile electric control of the spin-to-charge conversion^9^. In this context, the nonvolatile electric control of the reciprocal charge-to-spin conversion in these 2DEGs would be of great interest for developing reconfigurable SOT-MRAM and logic gates, offering the possibility to actively manipulate the torque by electric fields, and thus to design new architectures. The direction and amplitude of the spin accumulation is the key parameter in the design of SOT applications, as it determines the direction of magnetization switching. In conventional SOT devices based on HM/FM heterostructures, however, the relative direction between the injected current and the generated spin accumulation is fixed by the material used^10^, inhibiting any dynamical reconfiguration. This feature results in two main limitations in present-day SOT-MRAMs. First, the fixed direction of the SOT torque requires an inversion of polarity for the applied current in order to switch between the two magnetization states, which requires additional MRAM circuitry^11^. Second, the fixed direction and magnitude of the SOT torque prevents the selective switching of multiple bits sharing the same write layer in conventional SOT-MRAMs, inhibiting any in-memory logic operations. Achieving the nonvolatile electric control of the SOT could thus open the way to a disruptive generation of magnetic memories and logic devices, with high-density integration and dynamical reconfigurability. Several approaches have been pursued in this direction, based on the combination of spin Hall effect heavy metal/ferromagnetic heterostructures with piezoelectric^12^, ferroelectric^13,14^, and oxide materials^15–17^ or the replacement of the heavy metal by magnetically doped topological insulators^18^ (TIs). However, none of the above methods provide nonvolatile control of SOT together with efficient dynamical inversion of the SOT direction in a structure compatible with magnetic tunnel junction integration.

In this work, we propose to use 2DEGs to obtain the nonvolatile gate electric-field control of SOTs. An electric modulation of the 2DEG at a SrTiO_3_/Ta interface is achieved, providing two remanent and switchable resistivity states of the devices, with a large contrast of 1064%. We report the observation of sizable SOTs in a SrTiO_3_/metal system, and we further show that the SOT strength in this system can be modulated by the gate voltage with non-volatility, an inverted hysteresis being observed in the SOT dependence with the gate-electric field. This nonvolatile control of the SOT occurs over several orders of magnitude, down to full extinction and sign inversion. We also demonstrate the dynamical control of the SOT direction by the application of voltage pulses. The modulation of the SOT is found to originate from the combination of the gate dependence of the 2DEG band structure with the electric modulation of the current injection in the 2DEG.

## Results

### Magnetotransport properties

**Fig. 1:**
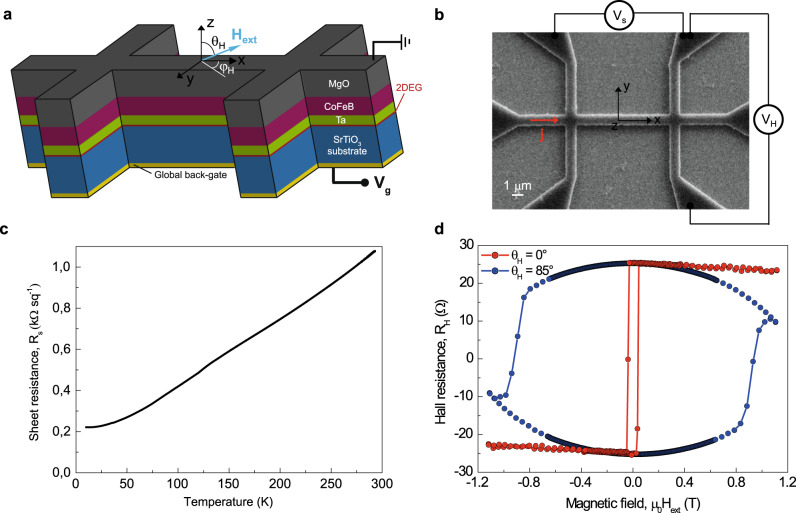
Schematic of the sample structure and its magnetotransport properties. **a** 3D schematic of the sample and of the coordinate system. **b** Scanning electron micrograph of a Hall cross-bar and measurement principle. **c** Temperature dependence of the stack sheet resistance *R*_s_. **d** Out-of-plane (*θ*_H_ = 0°) and near in-plane (*θ*_H_ = 85°, φ_H_ = 0°) magnetic field dependence of the Hall resistance at 10 K, showing well- defined up and down perpendicular magnetization states. All data have been measured with a 2D current density of *j* = 0.1 A cm^−1^ (*I*_0_ = 1 µA).

Experiments were carried out on Ta(0.9 nm)\CoFeB(0.9 nm)\MgO(1.8 nm)\Ta(1 nm) stacks deposited onto 500 μm (001)-oriented SrTiO_3_ substrates (see “Methods” for details on the sample preparation), allowing the creation of a 2DEG at the SrTiO_3_/Ta interface^19^. The 2DEG originates from the change of oxidation state of Ti atoms at the vicinity of SrTiO_3_ interface, from Ti^4+^ to Ti^3+^ due to oxygen vacancies (Ti^3+^ acting as electron donors). The development of 2DEGs using Ta\CoFeB\MgO stacks was motivated by three key points with respect to our earlier studies based on Al/NiFe:^8,9^ (i) the large interfacial magnetic anisotropy of the CoFeB\MgO interface^20^, that ensures perpendicular magnetization to the CoFeB ferromagnetic layer^21^, (ii) the lower resistance of TaOx^22^ for higher spin transparency, and (iii) the compatibility of Ta\CoFeB\MgO heterostructures for integration in magnetic tunnel junctions with high tunnel magnetoresistance^23^. The stack was patterned into 200 nm–1 μm wide and 2–10 μm long Hall cross-bars, as shown in Fig. 1a. To characterize the magnetic and electrical properties, an input current of magnitude up to 600 μA was injected along the *x* direction (Fig. 1b), and the longitudinal *R*_s_ and transverse Hall resistances *R*_H_ were measured as a function of the magnetic field ${{{H}}}_{{{{{{\rm{ext}}}}}}}$, which was applied along a direction defined by the polar and azimuthal coordinates *θ*_H_ and φ_H_. Hereafter, we present results obtained from 1 μm wide Hall bars, and define the injected linear current density as ${{j}}={{I}}/{{w}}$ (where *w* is the width of the Hall bar).

The temperature dependence of the sheet resistance of the device is shown in Fig. 1c for *j* = 0.1 A cm^−1^ (${{{I}}}_{0}=$1 µA). A reduction of the resistance by 380% is observed as the temperature decreases, which is characteristic of 2DEG conduction^8^. As shown in Fig. 1d, the anomalous Hall resistance follows a square-shaped magnetic hysteresis loop with applied out-of-plane magnetic field, indicating that the CoFeB has a perpendicular magnetization with 100% remanence. Furthermore, a reversible decrease of the Hall resistance is observed when increasing the in-plane field, indicating a coherent rotation of the CoFeB magnetization toward the hard plane direction. From the Hall resistance ${{{R}}}_{{{{{{\rm{H}}}}}}}={{{R}}}_{{{{{{\rm{AHE}}}}}}}{{\cos }}({{\theta }})+{{{R}}}_{{{{{{\rm{PHE}}}}}}}{{{\sin }}}^{2}({{\theta }})\,{{\sin }}(2{{\varphi }})$, where *θ* and φ are the polar and azimuthal angles of the magnetization in spherical coordinates, we determine the anomalous Hall *R*_AHE_ = 25.3 Ω and planar Hall *R*_PHE_ = 1.7 Ω resistances in the ungated state (see Supplementary Section S1). Note that an anomalous Hall resistance significantly larger than that of standard SiO_2_\Ta\CoFeB\MgO SOT devices^24, 25^ is achieved in our system, taking advantage of the use of ultra-thin Tantalum layer, as previously observed by Zhu et al.^26^.

### Nonvolatile electric control of the 2DEG properties

**Fig. 2:**
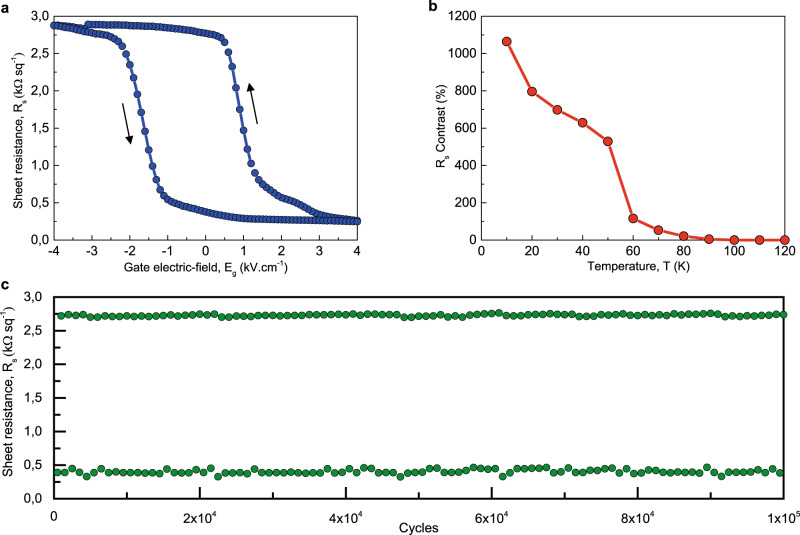
Nonvolatile electric-field control of the 2D gas properties. **a** Sheet resistance *R*_s_ as a function of the applied gate-electric field *E*_g_ across the sample, showing high and low-resistivity states of the device with *R*_s_ contrast of 1064%. The gate electric-field sequence is [+4, − 4, +4] kV cm^−1^ ([+200 V, −200V, + 200 V]), with the low-resistance state as the initial state. **b** Temperature dependence of the *R*_s_ contrast. **c** Endurance property of *R*_s_ at remanence (*E*_g_ = 0 kV cm^−1^) after application of positive and negative gate electric-field pulses of ±4 kV cm^−1^. All data have been measured at 10 K with a 2D current density of j = 0.1 A cm^−1^ (*I*_0_ = 1 µA), and after initialization of the ferromagnet in the up magnetization state.

To modulate the 2DEG properties, an electric field *E*_g_ is applied across the SrTiO_3_ substrate using a back-gate. Figure 2a shows the sheet resistance *R*_s_ as a function of the applied gate-electric field, at the temperature of 10 K and after initializing the ferromagnet in the up magnetization state. Hysteresis is observed in the sheet resistance, with two switchable and remanent high and low-resistivity states of the device. The R_s_ contrast, defined as (*R*_s,max_−*R*_s,min_)/*R*_s,min_, shows a value of 1064%, with 615% remanent contrast at *E*_g_ = 0 kV cm^−1^ between the high and low remanent resistivity states.

The hysteresis is inverted, i.e., it is anticlockwise, which is a typical characteristic of charge trapping effects^27^. It contrasts with previous results on 2DEGs at the SrTiO_3_\\Al interface, showing no hysteresis for 500 µm-thick SrTiO_3_ substrates^8^, and a clockwise loop compatible with field-induced ferroelectricity for thinner SrTiO_3_\\Al substrates^9^ (in which larger electric fields can be applied), as well as for ferroelectric Ca:SrTiO_3_\\Al system^28^. Here, the inverted loop is a clear indication of charge trapping^29^, and leads to a much larger resistance contrast. Similar inverted hysteresis were also evidenced in SrTiO_3_\\Metal\CoFeB\MgO 2DEG samples with different metal layers, as detailed in Supplementary Section S2. The oxidation of the metallic layer (here tantalum) creates oxygen vacancies at the surface of the TiO_2_-terminated SrTiO_3_ substrate, with a density enhanced by the annealing process at 300 °C. When applying a positive gate-electric field, the 2DEG is enriched in electrons, until reaching a saturated low 2DEG resistivity state. Some electrons gets trapped within the SrTiO_3_, very probably on oxygen vacancies located at the vicinity of the 2DEG^29, 30^. Because of these trapped electrons, the 2DEG is easily depleted when the gate-electric field is swept from positive to negative. This triggers a sharp increase of the resistivity of the 2DEG, the channel being almost completely depleted upon crossing zero field. Decreasing further the electric field toward negative values reinforce the depletion of the 2DEG, leading to a saturating high 2DEG resistivity state, and the detrapping of electrons. As the gate-electric field is reversely swept from negative to positive, the 2DEG is enriched in electrons, reaching the high conductive state again at zero field. As a result, the electron trapping and detrapping effects between the 2DEG and the SrTiO_3_ drives the nonvolatile behavior of the 2DEG conductivity.

As seen in Fig. 2b, the *R*_s_ contrast decreases upon increasing the temperature until full extinction of the Rs contrast at about 105 K, corresponding to the antiferrodistortive transition from cubic to the tetragonal phase of SrTiO_3_^31^, in agreement with others charge trapping studies in SrTiO3-based 2DEG^30^. The dynamic switching between the high and low-resistivity states was studied using a sequence of pulsed gate-electric field. The endurance of the device after application of successive positive and negative 200-ms-pulses is shown in Fig. 2c. The switching between stable high- and low-resistivity states shows a constant contrast, and no sign of fatigue within the 10^5^ cycle attempts. This confirms the technological potential of the charge trapping-related hysteretic effect observed here to control the 2DEG properties.

### Spin–orbit–torques characterization

We used the harmonic Hall voltage measurements method to quantify the spin–orbit torques in our systems^3,32,33^, An AC current of frequency ω/2π = 60 Hz and amplitude ${{{I}}}_{0}=$40 µA (*j* = 4 A cm^−1^) is injected along the *x* direction to induce small oscillations of the magnetization around its equilibrium. These oscillations generate first and second-harmonic contributions to the Hall resistance ${{{R}}}_{{{{{{\rm{H}}}}}}}({{{{{\rm{\omega }}}}}})={{{V}}}_{{{{{{\rm{H}}}}}}}({{{{{\rm{\omega }}}}}})/{{{I}}}_{0}={{{R}}}_{{{{{{\rm{H}}}}}},{{{{{\rm{\omega }}}}}}}+{{{R}}}_{{{{{{\rm{H}}}}}},2{{{{{\rm{\omega }}}}}}}$, providing a sensitive way to measure the current-induced fields. The first harmonic ${{{R}}}_{{{{{{\rm{H}}}}}},{{{{{\rm{\omega }}}}}}}$ corresponds to the Hall resistance measured in DC, while the second-harmonic term ${{{R}}}_{{{{{{\rm{H}}}}}},2{{{{{\rm{\omega }}}}}}}$ includes modulation of the Hall resistance by the SOT effective fields, ${{{R}}}_{{{{{{\rm{SOT}}}}}},2{{{{{\rm{\omega }}}}}}}$, as well as magneto thermal effect due to unintentional Joule heating, ${{{R}}}_{{{{{{\rm{T}}}}}},2{{{{{\rm{\omega }}}}}}}$. After subtracting longitudinal and perpendicular thermal effects contribution to ${{{R}}}_{{{{{{\rm{H}}}}}},2{{{{{\rm{\omega }}}}}}}$, SOT effective fields can be determined by sweeping the magnetic field along the *x* (*y*) direction. In the small angles approximation, one measures a longitudinal (transverse) SOT effective fields Δ*H*_x_ (Δ*H*_y_), respectively:^32^

1 $$\Delta {{{H}}}_{{{{{{\rm{x}}}}}}\left({{{{{\rm{y}}}}}}\right)}=\left(\frac{{{{{{\rm{d}}}}}}{{{R}}}_{{{{{{\rm{SOT}}}}}},2{{{{{\rm{\omega }}}}}}}}{{{{{{\rm{d}}}}}}{{{H}}}_{{{{{{\rm{x}}}}}}\left({{{{{\rm{y}}}}}}\right)}}\right)/\left(\frac{{{{{{{\rm{d}}}}}}}^{2}{{{R}}}_{{{{{{\rm{H}}}}}},{{{{{\rm{\omega }}}}}}}}{{{{{{\rm{d}}}}}}{{{H}}}_{{{{{{\rm{x}}}}}}\left({{{{{\rm{y}}}}}}\right)}^{2}}\right)$$

By defining ${{{{{\rm{\xi }}}}}}=\,{{{R}}}_{{{{{{\rm{PHE}}}}}}}/{{{R}}}_{{{{{{\rm{AHE}}}}}}}$, the anti-damping effective field *H*_AD_ and field-like effective field *H*_FL_ are given by:^32^

2 $${{{H}}}_{{{{{{\rm{AD}}}}}}\left({{{{{\rm{FL}}}}}}\right)}=-2\frac{\Delta {{{H}}}_{{{{{{\rm{x}}}}}}\left({{{{{\rm{y}}}}}}\right)}+2{{{{{\rm{\xi }}}}}}\Delta {{{H}}}_{{{{{{\rm{y}}}}}}\left({{{{{\rm{x}}}}}}\right)}}{1-4{{{{{{\rm{\xi }}}}}}}^{2}}$$

**Fig. 3:**
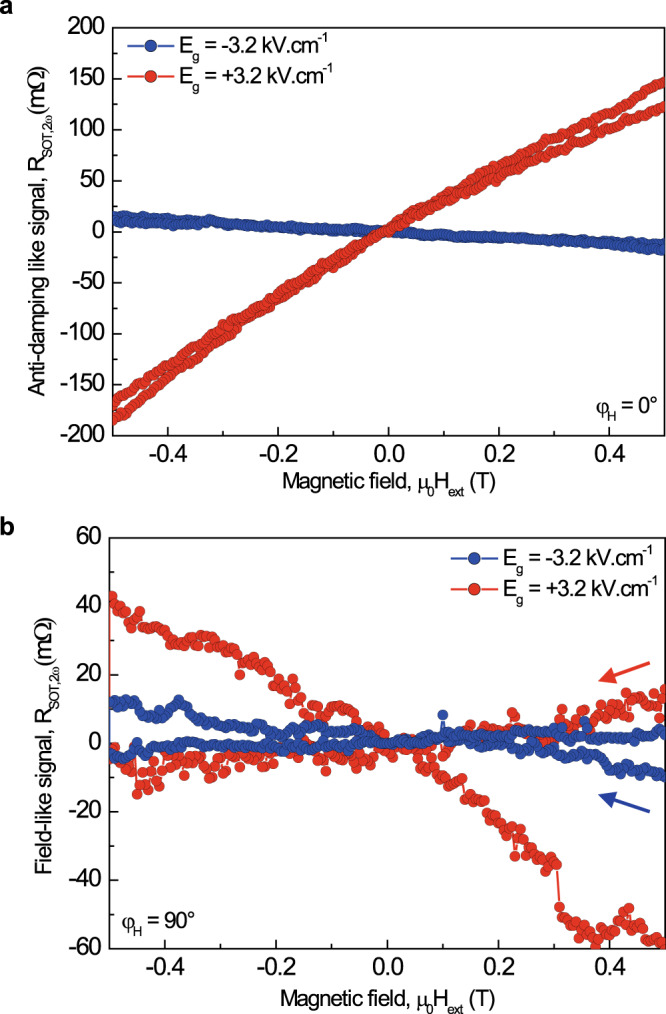
Spin–orbit torques characterization by second-harmonic Hall measurement. **a** Second-harmonic Hall resistances ${{{R}}}_{{{{{{\rm{SOT}}}}}},2{{{{{\rm{\omega }}}}}}}$ at φ_H_ = 0°, corresponding to the anti-damping like signal, for *E*_g_ = ±3.2 kV cm^−1^. **b** Second-harmonic Hall resistances ${{{R}}}_{{{{{{\rm{SOT}}}}}},2{{{{{\rm{\omega }}}}}}}$ at φ_H_ = 90°, corresponding to the field-like signal, for *E*_g_ = ±3.2 kV cm^−1^. All data have been measured at 10 K with a linear AC current density of j = 4 A cm^−1^ (*I*_0_ = 40 µA).

Measurements were performed with a magnetic field µ_0_*H*_ext_ applied close to the in-plane direction *(θ*_H_ = 85°, φ_H_ = 0, 90°) and swept between ± 1.1 T. Figure 3a, b shows the second-harmonic Hall resistances ${{{R}}}_{{{{{{\rm{SOT}}}}}},2{{{{{\rm{\omega }}}}}}}$ at φ_H_ = 0° and 90°, after subtraction of the thermal effects and experimental resistance offsets (see Supplementary Section S3), for positive and negative gate-electric fields of ±3.2 kV cm^−1^. Typical symmetric (antisymmetric) contributions are observed for the magnetic field perpendicular (parallel) to the current, corresponding to the field-like-torques and anti-damping like torques, respectively. Remarkably, we observe a sign change of ${{{{{{\rm{R}}}}}}}_{{{{{{\rm{SOT}}}}}},2{{{{{\rm{\omega }}}}}}}$ for high and low-resistivity states. When using Eqs. (1)–(2), we find µ_0_*H*_AD_ = +6.2 mT (−0.72 mT) and µ_0_*H*_FL_ = +1.35 mT (−0.39 mT) for the low (high) resistivity states measured at *E*_g_ = +3.2 kV cm^−1^ (−3.2 kV cm^−1^), respectively. Remarkably, the SOT effective fields are seen to be opposite for high and low-resistivity states, which we further discuss below through the study of the whole SOT electric-field dependence. It is worth noting that the amplitude of gate modulation of the field-like effective field is close to the measurement resolution limit. Hereafter, we focus on the anti-damping like torque, which is the important term for spin–orbit memory applications.

### Nonvolatile electric-field control of the spin–orbit torques

**Fig. 4:**
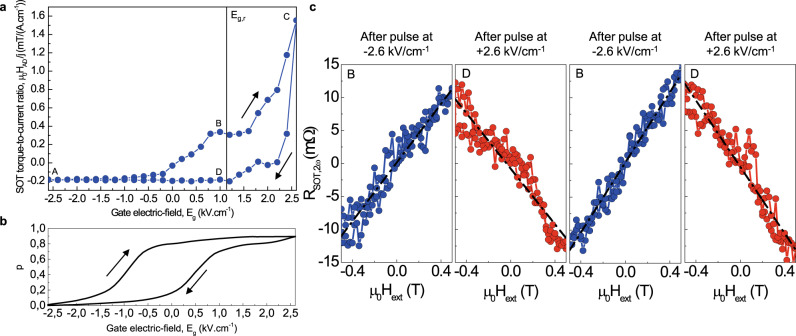
Nonvolatile electric-field control of the spin–orbit torques. **a** Gate electric-field dependence of the spin–orbit torques anti-damping-like effective field µ_0_*H*_AD_. The points A–D are examined in (**c**). **b** Measured portion *p* of the current injected in the 2DEG as a function of the gate-electric-field *E*_g_. **c** Normalized second-harmonic Hall resistance at shifted electrical remanence *E*_g,r_ after successive application of negative (blue) or positive (red) gate-electric-field pulses of ±2.6 kV cm^−1^. Dashed and dotted lines are linear fits, yielding SOT effective fields µ_0_*H*_AD_/*j* = +0.29, −0.20, +0.32, and −0.18 mT/(A cm^−1^), respectively. All data have been measured at 10 K with a linear AC current density of *j* = 4 A cm^−1^ (*I*_0_ = 40 µA) and a nearly in-plane magnetic field (*θ*_H_ = 85°, φ_H_ = 45°).

We now repeat SOT measurements described in the previous section while modulating the 2DEG properties with gate-electric fields ranging between ± 2.6 kV cm^−1^. Figure 4a shows the measured SOT anti-damping like effective field per linear current density µ_0_*H*_AD_/*j*, as a function of the gate-electric field. The results show a remanent modulation of the SOT-AD effective field, with inversion of the *H*_AD_ sign for opposite maximum gate-electric fields. The dependence shows a hysteresis that is inverted, similar to the resistance hysteresis shown in Fig. 2a.

Let us discuss the origin of this SOT hysteresis. The main contribution to this loop is the nonvolatile modulation of the 2DEG resistivity and thus of the current injection in the 2DEG. Figure 4b shows the measured modulation of the current injection in the 2DEG. The 2DEG and the Ta layer constitute two parallel channels of conduction with two separate SOT contributions. At maximum negative electric field (A), the device is in a high resistivity state which corresponds to the resistivity of the partially oxidized CoFeB/Ta bilayer (see Supplementary Section S4), indicating that the 2DEG is largely depleted and has an ultra-low conductance. Hence, there is no current in the 2DEG at this negative extrema. Inversely, the 2DEG resistivity reaches 0.26 kΩ sq^−1^ for the maximum positive electric field (C). The device is then in the low-resistivity state, which means that 92% of the applied current is flowing in the 2DEG.

A negative SOT-AD effective field of −0.23 mT/(A cm^−1^) is observed at the negative end of the sweep (A) when there is no current flowing in the 2DEG. This suggests that the SOT is then due to a residual contribution from the Ta layer, this interpretation being in good agreement, both in sign and magnitude, with previous observations on SiO_2_/Ta/CoFeB/MgO^24^. The highest SOT efficiency is achieved at the positive end of the electric-field sweep (C), when the 2DEG is in the low-resistivity state, reaching +1.6 mT/(A cm^−1^). Notably, the SOT-AD effective field at this extrema is positive, of opposite sign compared to that of the Ta/CoFeB/MgO system^24^ (see Supplementary Section S4). This confirms that when the device is in the low-resistivity state, the SOT arises from the 2DEG via the Edelstein effect, rather than being due to a residual Spin Hall effect in the Tantalum layer.

If the nonvolatile modulation of the 2DEG resistivity was the sole origin of the SOT hysteresis, one would expect to have similar hysteresis loops for the SOT (Fig. 4a) and for the resistance (Fig. 4b). However, the SOT hysteresis of Fig. 4a does not simply reflect the charge current redistribution within the stack. The differences between the loops of Fig. 4a and b are very probably due to the dependence of the spin–orbit conversion with the position of the Fermi level, which is linked to specific points of the k space. This has been previously studied in SrTiO_3_\Al^8^ and SrTiO_3_\LAO^6^ samples, which have shown different gate dependence in SrTiO_3_/Metal 2DEG systems depending on metal and sample preparation^19^. For a given current density flowing in the 2DEG, the produced SOT is expected to vary with the applied electric field. The SOT hysteresis has thus to be understood as resulting from both the current redistribution in the stack when varying the 2DEG resistivity, and the variations of the conversion with the 2DEG Fermi level position (see Supplementary Section S5).

The dynamical control of the SOT-AD effective field is further evidenced in Fig. 4c, which displays the measured normalized second-harmonic Hall resistance at shifted electrical remanence *E*_g,r_, after application of negative and positive 200-ms-long gate-electric-fields pulses of ±2.6 kV cm^−1^. A gate electric-field pulse is first applied to the device, then the first and second-harmonic Hall resistances are simultaneously measured at *E*_g,r_. The reproducible inversion of the SOT torque sign is demonstrated, achieving µ_0_*H*_AD_/*j* = +0.30 ± 0.1 mT/(A cm^−1^) and −0.19 ± 0.1 mT/(A cm^−1^) after negative and positive voltage pulses, respectively. The control of the SOT shows a deviation of less than ±0.1 mT/(A cm^−1^) over successive trials. For applications, it would be interesting to have a large remanence of the SOT at zero electric field. Here, the experiment was not performed at zero electric field due to the small SOT difference induced by intrinsic electric-field dependence of the band structure in our SrTiO_3_/Ta system, but at *E*_g,r,_ where the points B and D of Fig. 4a are well splitted. Note, however, that the intrinsic electric-field dependence of the band structure strongly dependent on the stack materials, as observed by Vicente-Arche et al.^19^ for different SrTiO_3_/Metal system. Hence, further material engineering can be made to ensure a large contrast at zero-field remanence for applications.

### Current and temperature dependence of the SOTs

**Fig. 5:**
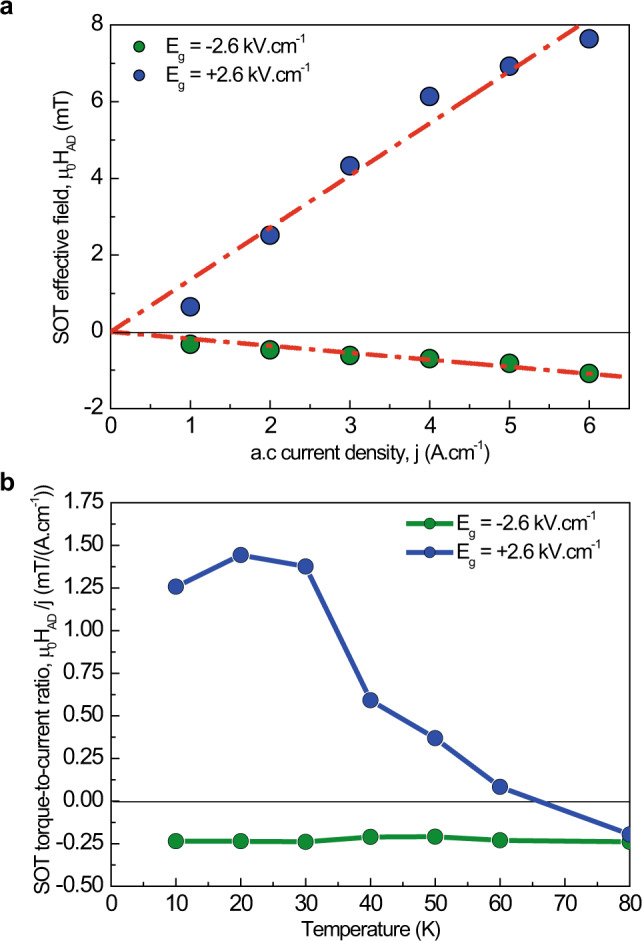
Dependence of the spin–orbit torques anti-damping like components on the injected current density and on the temperature. **a** Anti-damping like effective field µ_0_*H*_AD_ as a function of the applied 2D current density for positive (blue) and negative (green) gate electric-field of ± 2.6 kV cm^−1^ at the temperature of 10 K. Dashed and dotted lines are linear fits, yielding SOT effective fields µ_0_*H*_AD_ = +1.36 mT/(A cm^−1^) (−0.18 mT/(A cm^−1^)) for positive (negative) gate electric-field. **b**, Temperature dependence of the SOT anti-damping like effective field µ_0_*H*_AD_ at *j* = 2 A cm^−1^ for positive (blue) and negative (green) gate electric-field of ± 2.6 kV cm^−1^. All data have been measured with a nearly in-plane applied magnetic field (*θ*_H_ = 85°, φ_H_ = 45°).

The SOT characterizations were repeated for linear current densities ranging from 1 to 6 A cm^−1^ (*I*_0_ = 100–600 µA), for the device in the high (*E*_*g*_ = −2.6 kV cm^−1^) and low (*E*_g_ = +2.6 kV cm^−1^) resistivity states. Figure 5a shows that the amplitude of the SOT-AD effective field scales linearly with the linear current density, with a positive (negative) slope for the low (high) resistivity states, respectively. The linear scaling behavior confirms that Joule heating has a negligible impact on magnetic properties within the current range used for characterization^3^. This result further demonstrates that the SOT sign inversion is reproducible, with a difference as large as 8.7 mT at a current density of 6 A cm^−1^. Note that if the present study focuses on the quantitative measurement of the SOT modulation, both in sign and amplitude, it opens the way to magnetization-switching experiments. The device developed in this work presents large perpendicular magnetic anisotropy, together with a moderate SOT efficiency, preventing to perform magnetization switching at a current compatible with low-temperature operation. To obtain magnetization switching, several optimizations can be made, including increasing the thickness of CoFeB layer to reduce the perpendicular magnetic anisotropy, and optimizing the material and thickness of underlayer material (here Ta) for higher spin transparency and SOT efficiency.

Finally, the temperature dependence of µ_0_*H*_AD_*/j* in the high- and low-resistivity states is shown in Fig. 5b. In the high resistivity state (*E*_g_ = −2.6 kV cm^−1^), the result shows a constant AD-SOT torque-to-current ratio of – 0.23 mT/(A cm^−1^), with a less than ±0.01 mT/(A cm^−1^) deviation in the 10 K to 80 K temperature range. This is in good agreement with our hypothesis of a current injected only in the Ta layer, generating a SOT which is expected to be independent of the temperature^24^.

At the opposite gate electric-field (*E*_g_ = −2.6 kV cm^−1^), µ_0_*H*_AD_/*j* is found constant from 10 to 30 K range, at around +1.35 ± 0.10 mT/(A cm^−1^), followed by a decrease with increasing temperatures, reaching −0.23 mT/(A cm^−1^) at 80 K. Notably, the AD-SOT torque-to-current ratio at this temperature of 80 K is identical for *E*_g_ = ±2.6 kV cm^−1^, suggesting a common origin. This result is in good agreement with the measured *R*_s_ contrast displayed in Fig. 2b, in which we observed a merging of the high and low-resistivity states above 80 K. Above this temperature range, the conductivity in these 2DEGs is known to disappear^19^, and thus the current mostly flows in the Ta layer, which becomes the only SOT generator.

To conclude, the nonvolatile electric control of the spin–orbit torques in 2DEGs could open the way to a new generation of spin–orbit torques devices. Concerning memory applications, the additional functionality provided by the electric control can be used for building reconfigurable SOT-MRAM, in novel architectures suitable for efficient and fast operation. While efforts remains to be made to bring this technology to room temperature, the development of CoFeB/MgO heterostructures on SrTiO_3_ with strong CoFeB perpendicular magnetization ensures compatibility for future integration in magnetic tunnel junctions. Beyond memory applications, this nonvolatile control of SOTs offers an additional way to manipulate skyrmions, domain walls or magnons by permitting a local control of the SOT along circuits. It could also be used in SOT oscillators, to allow developing logic architectures and agile terahertz emitters.

## Methods

The Ta(0.9 nm)/CoFeB(0.9 nm)/MgO(1.8 nm)/Ta(1 nm) samples have been deposited by DC magnetron sputtering on TiO_2_-terminated (001)-oriented SrTiO_3_ substrates of 500 μm thickness (from SurfaceNet). TiO_2_-termination was achieved through a chemical treatment, where the substrate was subsequently submerged in H_2_O for 10 min and an acid solution (HCl 3: HNO_3_ 1: H_2_O 16) for 20 min. The Hall bar devices were patterned by electron-beam lithography into 200–1000 nm-wide crosses, and subsequent deposition and lift off of the stack. After deposition, the stack was annealed at 300 °C during 10 min to crystallize the MgO. A sample-wide back-gate of Ti(10 nm)/Au(100 nm) was then added by evaporation. The Hall voltage measurements were performed by using an AC current with an amplitude of 200–600 μA, modulated at ω/2π = 60 Hz. *V*_H,ω_ and *V*_H,2ω_ were recorded simultaneously using 2 lock-ins during sweeps of the external magnetic field for 6 s at each field step.

### Reporting summary

Further information on research design is available in the Nature Portfolio Reporting Summary linked to this article.

## Supplementary information

Supplementary Information

Reporting Summary

## Data Availability

All data generated in this study have been deposited in a public database under the accession code 10.57745/IZHDPC.
