# Supplementary material for: Non-volatile electric control of spin-orbit torques in an oxide two-dimensional electron gas
Source: Nat Commun. 2023 May 5;14:2590. doi: 10.1038/s41467-023-37866-2 (PMC10162979; doi:10.1038/s41467-023-37866-2)
Supplement: Supplementary file 1 — Supplementary Information [file 41467_2023_37866_MOESM1_ESM.pdf]

Supplementary material

Non-volatile electric control of spin-orbit torques in an oxide two-dimensional electron gas

Cécile Grezes[1], Aurélie Kandazoglou[1], Maxen Cosset-Cheneau[1†], Luis M. Vicente Arche[2], Paul Noël[1,3], Paolo Sgarro[1], Stephane Auffret[1], Kevin Garello[1], Manuel Bibes[2], Laurent Vila[1*], and Jean-Philippe Attané[1,4*]

[1]Université Grenoble Alpes / CEA / IRIG/ SPINTEC, Grenoble, France
[2]Unité Mixte de Physique, CNRS, Thales, Université Paris-Saclay, Palaiseau, France
[3]Department of Materials, ETH Zurich, 8093 Zurich, Switzerland
[4]Institut Universitaire de France
[†]Current address: Physics of Nanodevices, Zernike Institute for Advanced Materials, University of Groningen, 9747 AG Groningen, Netherlands
* e-mail : jean-philippe.attane@cea.fr, laurent.vila@cea.fr

Table of Contents:

S1. Determination of the AHE and PHE contributions

S2. Electric-control of 2DEGs with anticlockwise hysteresis in SrTiO3\\Metal\CoFeB\MgO

S3. Separating SOT effective fields from thermal contributions

S4. SiO_2\\Ta\CoFeB\MgO reference sample characterization

S5. SOT contributions from the 2DEG and the Tantalum layer

S1. Determination of the AHE and PHE contributions

The AHE and PHE contributions have to be measured to quantify the SOT effective fields using Eq. (2). This is achieved by measuring the first harmonic Hall resistance, as a function of the external magnetic field applied almost in-plane (θ_B = 85°) and at an angle $\varphi_H \neq$ 0º, 90º. Fig. S1 shows $R_{H,\omega}$ as a function of $\mu_0 H_{ext}$ for an angle φ_H = 45° in the ungated state as an example. As AHE and PHE are respectively odd and even with respect to magnetization reversal, $R_{AHE,\omega}$ and $R_{PHE,\omega}$ can be separated by antisymmetrization and symmetrization of $R_{H,\omega}$, respectively. Fig. S1b (Fig. S1c) shows the resulting $R_{AHE,\omega}$ ($R_{PHE,\omega}$) as a function of the external magnetic field (φ_H = 45°) for this example. The saturation value of the AHE resistance reads directly from the contrast of $R_{AHE,\omega}$, yielding R_{AHE} = 25.3 Ω. The PHE saturation resistance is deduced from the linear fit of $R_{PHE,\omega}$ as a function of $\sin^2(\theta)$ (Fig. S1d), yielding R_{PHE} = 1.7 Ω. The equilibrium angle of magnetization (θ_0) as function of the applied external magnetic field is further determined from the first
harmonic Hall measurement as [1] $\theta_0 = \arccos\left|\frac{R_{AHE,\omega}(\mu_0 H_{ext})}{R_{AHE}}\right|$.

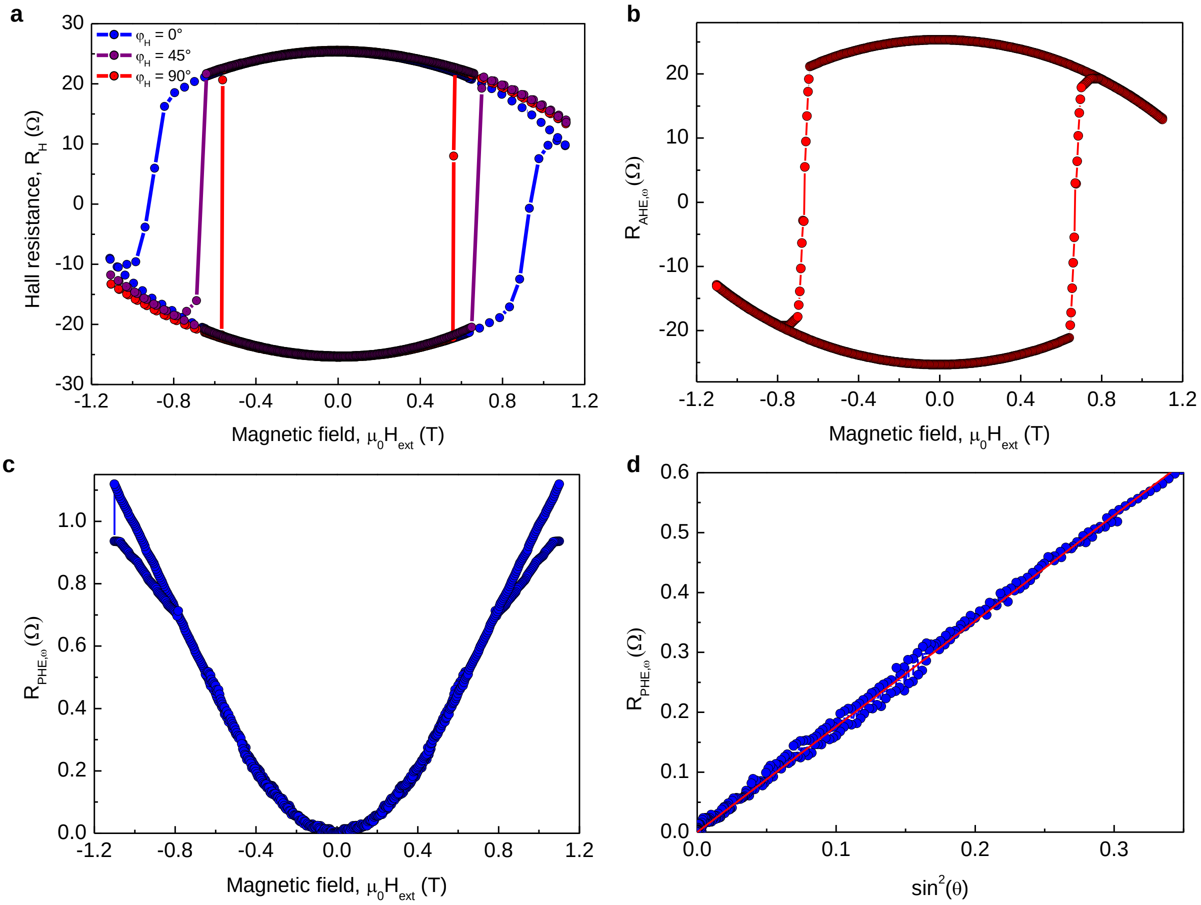

Figure S1 | Determination of the AHE and PHE contributions. a, First harmonic Hall resistance $R_{H,\omega}$ as a function of the magnetic field applied almost in-plane (θ_H = 85°, φ_H = 0°, 45°, 90°). **b,** Antisymmetric AHE signal $R_{AHE,\omega}$ of $R_{H,\omega}(H_{ext,45°})$ yielding R_{AHE} = 25.3 Ω. **c,** Symmetric PHE signal $R_{PHE,\omega}$ of $R_{H,\omega}(H_{ext,45°})$. **d,** $R_{PHE,\omega}$ as a function of $\sin^2(\theta)$ showing the expected linear dependence, the slope yielding the PHE resistance R_{PHE} = 1.7 Ω. The linear fit appears in red. All data have been measured in the ungated state at 10K with a 2D current density of j = 4 A.cm^{-1}.

S2. Electric-control of 2DEGs with anticlockwise hysteresis in $SrTiO_3$\\Metal\CoFeB\MgO

The electric-control of the 2DEG has been investigated in several $SrTiO_3$\\Metal\CoFeB\MgO samples with different metallic layers (Metal) in the scope of this study. Ta, Ru, Mg(0.9 nm)\CoFeB(0.9 nm)\MgO(1.8 nm)\Ta(1 nm) stacks were deposited onto 500 µm (001)-oriented $SrTiO_3$ substrates in order to create a 2DEG at the $SrTiO_3$/Metal interface, following an identical sample preparation (cf. § Methods). The stacks were patterned into 1 µm wide and 10 µm wide Hall bar devices by electron-beam lithography, and sample-wide back-gates of Ti(10 nm)/Au(100 nm) were deposited by evaporation. Fig. S2 shows the sheet resistance R_s as a function of the applied gate electric-field for $SrTiO_3$\\Mg\CoFeB\MgO and $SrTiO_3$\\Ru\CoFeB\MgO samples, at the temperature of 10 K and after initializing the ferromagnet in the up magnetization state. Similar anticlockwise hysteresis to

those seen in SrTiO$_3$\\Ta\CoFeB\MgO samples are observed, with two switchable and remanent high and low resistance states. This confirms the appearance of charge trapping effect at different SrTiO$_3$/Metal interface. The R_s contrast shows a value of 1598% (2%) for the SrTiO$_3$\\Mg\CoFeB\MgO (SrTiO$_3$\\Ru\CoFeB\MgO) sample. Notably, the R_s contrast of SrTiO$_3$\\Ru\CoFeB\MgO sample is significantly lower than that of SrTiO$_3$\\Ta,Mg\CoFeB\MgO samples, which probably indicates a poor creation of oxygen vacancies by the Ru layer at the surface of the TiO$_2$-terminated SrTiO$_3$ substrate. We note also the appearance of a shift of the hysteresis, which depends on the nature of the metallic layer.

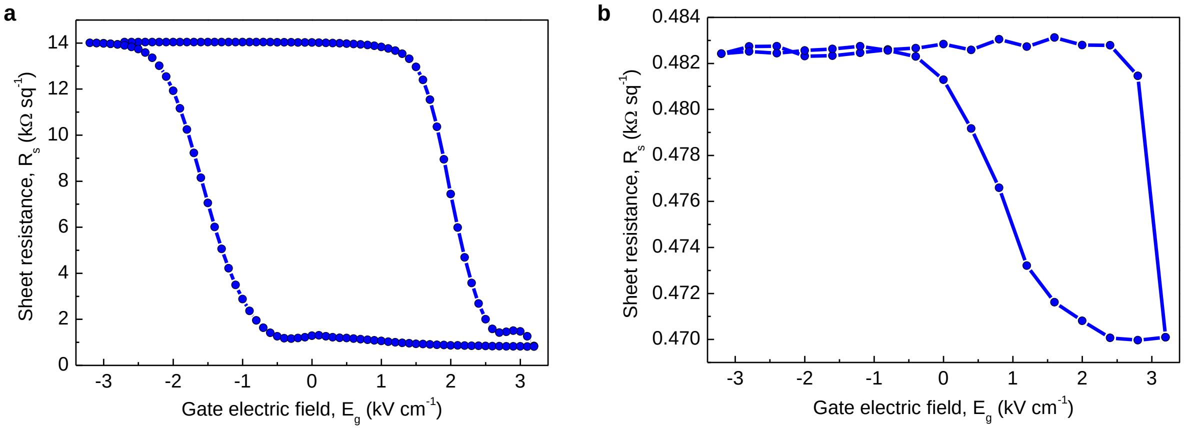

Figure S2 | Electric-control of 2DEGs in SrTiO3\\Mg\CoFeB\MgO and SrTiO3\\Ru\CoFeB\MgO samples. Sheet resistance R_s as a function of the applied gate electric-field E_g across the sample, showing anticlockwise hysteresis with high and low resistivity states of **a,** SrTiO$_3$\\Mg\CoFeB\MgO and **b,** SrTiO$_3$\\Ru\CoFeB\MgO Hall bar devices. All data have been measured at 10K, with a 2D current density of j = 0.1 A.cm^{-1}.

S3. Separating SOT effective fields from thermal contributions

To determine the SOT effective fields, the second harmonic Hall resistance $R_{H,2\omega}$ must be decorrelated from other contributions, corresponding to the magneto thermal contributions and to the effect of voltage probes misalignment [1] [2]. The ac current flowing into the Hall bar generates a modulated temperature gradient, which induces a second harmonic component to the Hall voltage via two types of thermoelectric voltages: the Seebeck effect and the Anomalous Nernst-Ettingshausen effect (ANE). The Seebeck effect, as well as the asymmetry of the voltage probes, induces a constant offset R_{Offset} in $R_{H,2\omega}$. The ANE, on the other hand, is perpendicular to the temperature gradient and to the magnetization easy axis, and induces a second harmonic contribution proportional to the AHE in $R_{H,2\omega}$. Fig. S3a (Fig. S3c) shows $R_{H,2\omega}$ as a function of $\mu_0 H_{ext}$ applied at $\theta_B = 85°$ and $\varphi_B = 0°$ (90°) for negative and positive gate electric-fields of ± 3.2kV.cm^{-1}. The ANE contribution (R_{ANE}) is observed as the difference of $R_{H,2\omega}$ measured at zero field for positive and negative sweeps of $\mu_0 H_{ext}$, together with a constant offset (R_{Offset}) for E_g = ± 3.2kV.cm^{-1} respectively. Both R_{Offset} and R_{ANE} can be subtracted from the raw data using [1]:

$$R_{SOT,2\omega} = R_{H,2\omega} - R_{ANE}\frac{R_{H,\omega}}{2R_{AHE}} - R_{Offset} \qquad (S1)$$

Fig. S3b (Fig. S3d) shows the resulting SOT anti-damping like signal (field-like signal) after subtraction of the thermal effects contributions R_{Offset} and R_{ANE}. The data shown are taken inside the hysteresis. The arrows indicate the sweeping field direction, emphasizing the inversion of the anti-damping-like torque direction under the two different gates. Inside the hysteresis, the magnetization direction (theta angle path) is different for the positive ($M_\uparrow$) and negative ($M_\downarrow$) sweeps of magnetic field. Consequently, its susceptibility to the field-like term is different inside the hysteresis, as magnetization follows a different path, which explains the curve opening for the field-like signal. Outside the hysteresis, the curves are well closing-up, as shown in Fig. S3c. Thermal signals arising from Nernst effect are well corrected, as evidenced by the crossing close to 0 at zero-field (for in-plane thermal gradient along current direction).

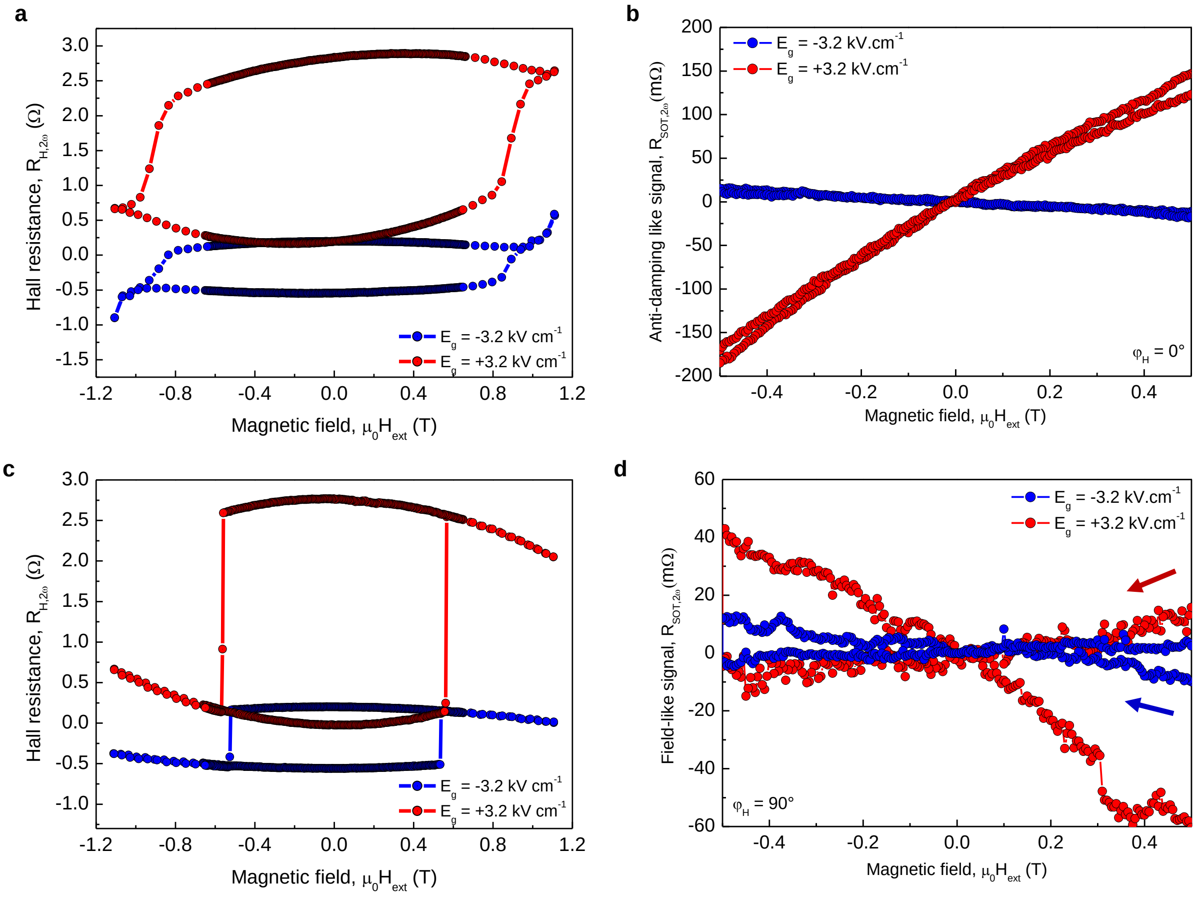

Figure S3 | Separating SOT effective fields from thermal contributions. a, Raw second harmonic Hall resistance $R_{H,2w}$ measured as a function of the magnetic field applied at θ_B = 85° and φ_B = 0° for negative (blue) and positive (red) gate electric-field of ± 3.2kV.cm^{-1}, and **b,** corresponding anti-damping like signal. **c,** Raw second harmonic Hall resistance $R_{H,2w}$ measured as a function of the magnetic field applied at θ_B = 85° and φ_B = 90° for negative (blue) and positive (red) gate electric-fields of ± 3.2kV.cm^{-1}, and **d,** corresponding field-like signal. All data have been measured at 10K with a 2D current density of j = 4 A.cm^{-1}.

S4. SiO_2\\Ta\CoFeB\MgO reference sample characterization

A SiO_2\\Ta\CoFeB\MgO reference sample has been prepared for comparison, with identical deposits to those made for the $SrTiO_3$\\Ta\CoFeB\MgO samples. The stack was patterned into 1 µm wide and 10 µm long Hall cross-bars for electrical measurements. An input current of magnitude 400 A was injected along the *x* direction, and the longitudinal R_s and transverse Hall resistances R_H were measured as a function of the external magnetic field $\mu_0 H_{ext}$. As shown in Fig. S4a, the temperature dependence of the sheet resistance of the device is characteristic of a metallic behavior, as expected from the metallic CoFeB/Ta bilayer, with a sheet resistance reaching 0.69 kΩ.sq^{-1} at 10K. This sheet resistance is significantly lower than that of the CoFeB/Ta bilayer in $SrTiO_3$\\Ta\CoFeB\MgO sample, confirming that the CoFeB/Ta bilayer is partially oxidized via oxygen reduction in contact of $SrTiO_3$.

Fig. S4b shows the anomalous Hall resistance as a function of the applied out-of-plane magnetic field. A square-shaped magnetic hysteresis loop is observed, indicating that the CoFeB has a perpendicular magnetization. To quantify the spin-orbit torques, harmonic Hall voltage measurements are performed with the magnetic field $\mu_0 H_{ext}$ applied almost in-plane (θ_H = 85°, φ_H = 0, 90°). Fig. S4 c and d shows the first and second harmonic Hall resistances $R_{H,\omega}$ and $R_{H,2\omega}$, respectively. Following the procedure described in the main text, the anti-damping like torques and field like-torques are determined after subtracting the thermal effects contribution to $R_{H,2\omega}$, yielding $\mu_0 H_{AD}/j$ = -1.5 mT/(A.cm^{-1}) and $\mu_0 H_{FL}/j$ = -0.43 mT/(A.cm^{-1}). Notably, the SOT-AD effective field of the SiO_2\\Ta\CoFeB\MgO reference sample is negative, of opposite sign compared to that of the $SrTiO_3$\\Ta\CoFeB\MgO system in the low 2DEG resistivity state. This confirms that the SOT arises from the 2DEG via the Edelstein effect for $SrTiO_3$\\Ta\CoFeB\MgO in low 2DEG resistivity state, rather than from a residual Spin Hall effect in the Tantalum layer. Note that the AD-SOT in the SiO_2\\Ta\CoFeB\MgO reference

sample is slightly higher than that of the $SrTiO_3$\\Ta\CoFeB\MgO samples in high resistivity state, in agreement with the partial oxidation of the CoFeB/Ta bilayer of the latter.

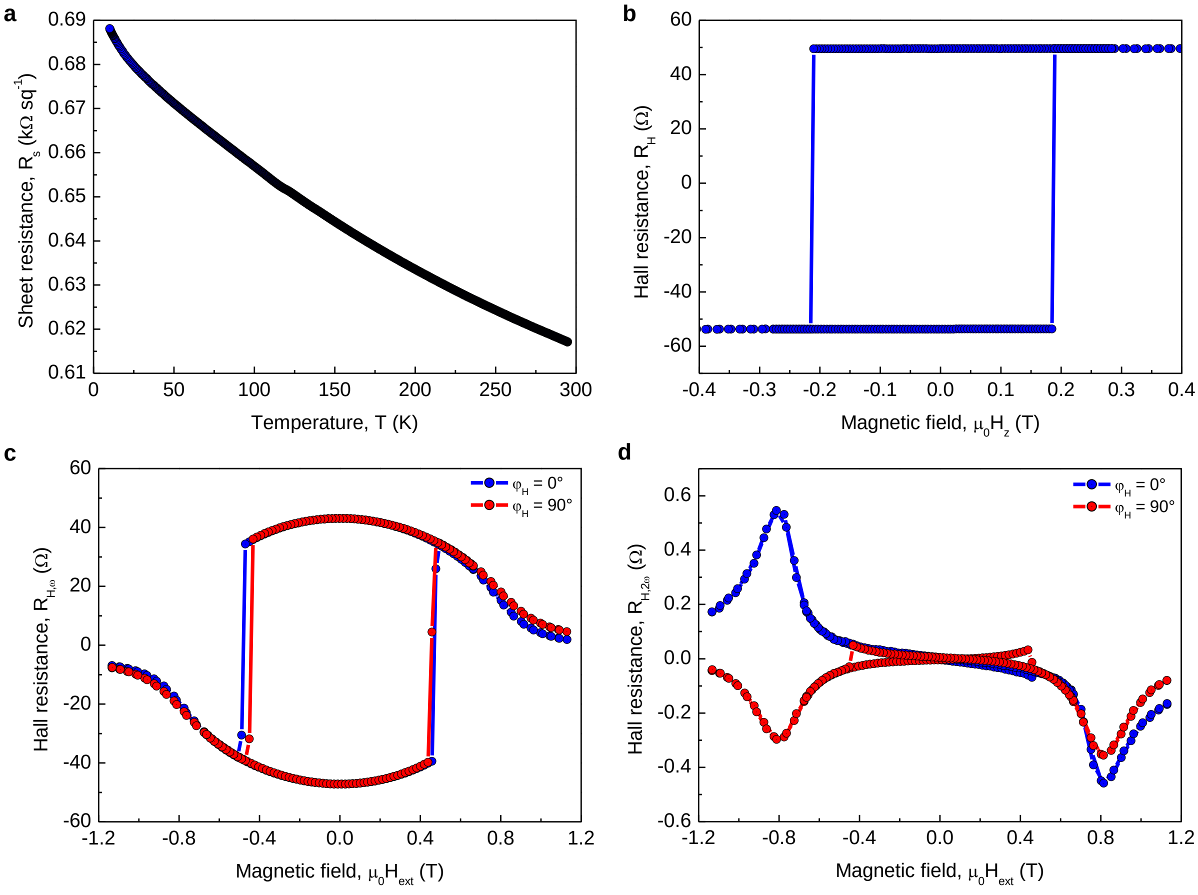

Figure S4 | SiO_2\\Ta\CoFeB\MgO reference sample. a, Temperature dependence of the stack sheet resistance R_S. **b,** Out-of-plane (θ_H = 90°) magnetic field dependence of the Hall resistance at 10 K, showing well-defined up and down perpendicularly magnetized states. **c,** First $R_{H,\omega}$ and **d,** second $R_{H,2\omega}$ harmonic Hall resistances as a function of the magnetic field applied almost in-plane (θ_B = 85°, φ_B = 0°, 90°) at 10K. All data have been measured with a 2D current density of j = 4 A.cm^{-1}.

S5. SOT contributions from the 2DEG and the Tantalum layer

The 2DEG and the Ta layer constitute two parallel channels of conduction, with two corresponding SOT contributions to the total SOT. In the following, we refer to $H_{AD,\,2DEG}$ ($H_{AD,Ta}$) the intrinsic SOT contribution from the 2DEG (Ta layer) alone. In a simple model, one can assume that the total SOT is the sum of the SOT contributions (in mT), weighted by the fraction of current injected in the corresponding conduction channels:

$$H_{AD,signal}=pH_{AD,2DEG}+(1-p)H_{AD,Ta}$$

where *p* is the fraction of current injected in the 2DEG, displayed in Fig. 4b, and $pH_{AD,2DEG}$ and $(1-p)H_{AD,Ta}$ the 2DEG-induced and Ta-induced anti-damping like SOT, respectively. The ratio of 2DEG-induced SOT to Ta-induced SOT is :

$$r_{AD}=\frac{pH_{AD,2DEG}}{(1-p)H_{AD,Ta}}=\frac{H_{AD,signal}-(1-p)H_{AD,Ta}}{(1-p)H_{AD,Ta}}$$

As a bulk metal, the intrinsic Ta-induced SOT $H_{AD,Ta}$ does not depend on the gate electric-field, and is revealed directly in the high resistivity state $H_{AD,\,Ta}=H_{AD,signal}(-E_{g,max})$ when there is no current in the 2DEG (i.e p=0) and where the SOT is constant. The left panel of the Figure R3 below shows the requested ratio r_{AD} of 2DEG-induced SOT to Ta-induced SOT at 10K, as a function of the gate electric-field. At large positive gates, the SOT is mostly due to the 2DEG, whose contribution is up to 82 times larger than that of the Ta layer. The variations of the overall SOT with the electric-field are due both to the current redistribution in the stack when varying the 2DEG resistivity, and to the variations of the conversion with the 2DEG Fermi level position. The temperature dependence of r_{AD} in the low resistivity state can be read directly from Fig. 5b with $H_{AD,\,Ta}(T)=H_{AD,signal}(-E_{g,max},\,T)$, and is displayed on the right panel (for j = 2 A. cm^{-1}).

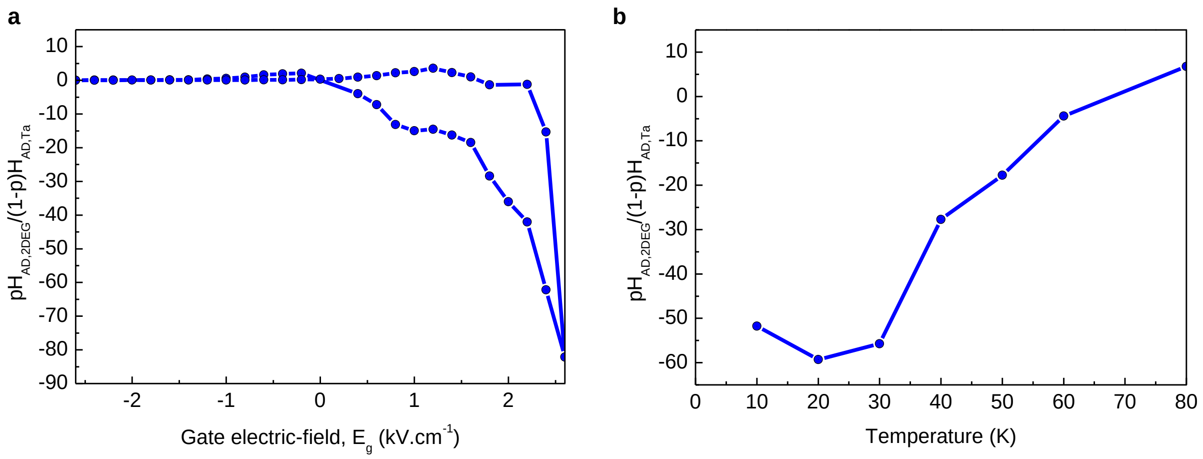

Figure S5 | 2DEG-induced SOT to Ta-induced SOT contributions. a, Ratio of 2DEG-induced SOT to Ta-induced SOT as a function of gate electric-field at the temperature of 10K and for j= 4 A.cm^{-1}**. b,** Temperature dependence of the ratio of 2DEG-induced SOT to Ta-induced SOT in the low resistivity state (E_g = +2.6 kV. cm^{-1}) for j= 2 A.cm^{-1}**.**

REFERENCES

[1] K. Garello et al., «Symmetry and magnitude of spin-orbit torques in ferromagnetic heterostructures,» *Nature Nanotechnology*, vol. 8, pp. 587-593, 2013.

[2] M. Hayashi et al., «Quantitative characterization of the spin-orbit torque using Harmonic Hall voltage measurements,» *Physical Review B*, vol. 89, n° 11144425, 2014.
